# At least three families of hyphosphere small secreted cysteine‐rich proteins can optimize surface properties to a moderately hydrophilic state suitable for fungal attachment

**DOI:** 10.1111/1462-2920.15413

**Published:** 2021-05-19

**Authors:** Zheng Zhao, Feng Cai, Renwei Gao, Mingyue Ding, Siqi Jiang, Peijie Chen, Guan Pang, Komal Chenthamara, Qirong Shen, Günseli Bayram Akcapinar, Irina S. Druzhinina

**Affiliations:** ^1^ Key Laboratory of Plant Immunity, Jiangsu Provincial Key Lab of Solid Organic Waste Utilization Nanjing Agricultural University Nanjing China; ^2^ Fungal Genomics Laboratory (FungiG) Nanjing Agricultural University Nanjing China; ^3^ Institute of Chemical, Environmental and Bioscience Engineering (ICEBE), TU Wien Vienna Austria; ^4^ Department of Medical Biotechnology, Institute of Health Sciences Acibadem Mehmet Ali Aydinlar University Istanbul Turkey

## Abstract

The secretomes of filamentous fungi contain a diversity of small secreted cysteine‐rich proteins (SSCPs) that have a variety of properties ranging from toxicity to surface activity. Some SSCPs are recognized by other organisms as indicators of fungal presence, but their function in fungi is not fully understood. We detected a new family of fungal surface‐active SSCPs (saSSCPs), here named hyphosphere proteins (HFSs). An evolutionary analysis of the HFSs in Pezizomycotina revealed a unique pattern of eight single cysteine residues (C‐CXXXC‐C‐C‐C‐C‐C) and a long evolutionary history of multiple gene duplications and ancient interfungal lateral gene transfers, suggesting their functional significance for fungi with different lifestyles. Interestingly, recombinantly produced saSSCPs from three families (HFSs, hydrophobins and cerato‐platanins) showed convergent surface‐modulating activity on glass and on poly(ethylene‐terephthalate), transforming their surfaces to a moderately hydrophilic state, which significantly favoured subsequent hyphal attachment. The addition of purified saSSCPs to the tomato rhizosphere had mixed effects on hyphal attachment to roots, while all tested saSSCPs had an adverse effect on plant growth *in vitro*. We propose that the exceptionally high diversity of saSSCPs in *Trichoderma* and other fungi evolved to efficiently condition various surfaces in the hyphosphere to a fungal‐beneficial state.

## Introduction

Recent studies have shown that besides enzymes and small molecules, the secretomes of filamentous and dimorphic fungi can frequently contain a diversity of small secreted cysteine‐rich proteins (SSCPs) unique for fungi (Kubicek *et al*., 2011, 2019; Wang *et al*., 2019; Feldman *et al*., 2020; Gao *et al*., 2020; Huber *et al*., 2020). This diverse group comprises a few distinct protein families, such as hydrophobins (HFBs) and cerato‐platanins (CPs), although most SSCPs remain unclassified. Individual fungal genomes can contain from a few dozens of such proteins in yeasts (Wang *et al*., 2019; Sun *et al*., 2020) up to several hundred in mushrooms (Zajc *et al*., 2013; Krizsán *et al*., 2019). The SSCPs are characterized by their small size (<300 amino acids, a.a.) (Pellegrin *et al*., 2015; Kubicek *et al*., 2019), the presence of a relatively short signal peptide (~20 a.a.) (Chen *et al*., 2013; Kim *et al*., 2016; Feldman *et al*., 2020) and cysteine enrichment. The latter feature is crucial for their properties and structure but is frequently ambiguous. For example, Kubicek *et al*. (2019) proposed a minimum threshold of 5% cysteine residues for SSCPs, referencing an average 1.5% content in all fungal proteins. Other authors rely on the presence of an evolutionarily conserved cysteine pattern in the primary structure, which can consist of only four residues in the CPs (Pazzagli *et al*., 1999; Chen *et al*., 2013; Gaderer *et al*., 2014) and up to eight in the HFBs (Wösten, 2001; Aimanianda *et al*., 2009; Bayry *et al*., 2012).

Probably the most interesting feature of SSCPs is their abundant secretion in liquid medium (up to 20%–30% of the total secretome) (Pellegrin *et al*., 2015; Feldman *et al*., 2020; Gao *et al*., 2020) and the unusually high polymorphism of their primary structures (Gaderer *et al*., 2014; Krizsán *et al*., 2019). Some SSCPs have a long evolutionary history of gene duplications (GDs) and lateral gene transfers (LGTs), making them frequent members of the orphomes (the genome fraction comprising orthologue‐less genes) of individual fungal taxa (Przylucka *et al*., 2017a; Feldman *et al*., 2019; Gao *et al*., 2020). Paradoxically, despite the abundance of SSCPs in fungal secretomes, functions have been proposed for only a few, while the role of the majority of them remains unknown (Aimanianda and Latge, 2010; Feldman *et al*., 2017; Wang *et al*., 2019; Gao *et al*., 2020).

Obviously, the diversity of SSCP structures corresponds to their functional versatility. Indeed, SSCPs are involved in all fungal life cycle stages and their environmental interactions (Grunbacher *et al*., 2014; Pellegrin *et al*., 2015; Feldman *et al*., 2017; Wang *et al*., 2019; Gao *et al*., 2020). Many are essential for specific stages of fungal reproduction and dispersal (Lugones *et al*., 1999; Lugones *et al*., 2004; Grunbacher *et al*., 2014; Cai *et al*., 2020) and for biotic interactions with plants (Frías *et al*., 2011; Guzmán‐Guzmán *et al*., 2017), animals (Andersson *et al*., 2013; Wang *et al*., 2019) and bacteria (Kombrink *et al*., 2019). SSCPs modulate fungal attachment and substrate colonization (Viterbo and Chet, 2006; Rosado *et al*., 2007; Quarantin *et al*., 2019), the stress response (Khalesi *et al*., 2016; Przylucka *et al*., 2017a) and general fungal fitness (Cai *et al*., 2020). However, numerous functional genetic studies based on the characterization of gene‐deletion mutants have demonstrated that they play only a minor or accessory role in such processes (Jeong *et al*., 2007; Frías *et al*., 2011; Frischmann *et al*., 2013), suggesting that the main forces driving and shaping SSCP diversity and evolution in fungi have yet to be revealed.

The large cluster of SSCP investigations reflects that other organisms use them as an indicator of fungal presence, probably because they are produced in large quantities. Therefore, SSCPs are frequently studied as effectors and elicitors, i.e. from the perspective of the host plants or animals in which the SSCPs either cause toxicity or elicit an immune response (Pellegrin *et al*., 2015; Dagvadorj *et al*., 2017; Darwiche *et al*., 2017; Fang *et al*., 2019; Li *et al*., 2019; Wang *et al*., 2019; Yu *et al*., 2020). These studies reveal the ecological role of individual SSCPs, but they do not sufficiently explain their function given that orthologous SSCPs are also present in saprotrophic fungi (Feldman *et al*., 2020; Gao *et al*., 2020).

The exceptionally high diversity of fungal SSCPs and their ubiquitous presence in biotrophic and saprotrophic fungi suggest their functions are related to aspects of the fungal life cycle other than host interactions (Garrigues *et al*., 2018; Hajdu *et al*., 2019; Holzknecht *et al*., 2020). The SSCP CPL1 in the human pathogenic yeast *Cryptococcus neoformans* (Tremellales, Basidiomycota) is important for its adhesion to and invasive growth into substrates (Sun *et al*., 2020). Similarly, at least two SSCP families in filamentous fungi (HFBs and CPs) are amphiphilic, having profound or even superior surface activity (Wösten, 2001; Gaderer *et al*., 2014) and the ability to reduce the force of water tension (van der Vegt *et al*., 1996). HFBs are particularly small (~100 a.a.) and have a conserved pattern of eight cysteine residues C‐CC‐C‐C‐CC‐C that efficiently stabilizes their tertiary structure by disulfide bonds (Linder *et al*., 2005). CPs are slightly larger (~150 a.a.) and have only four single cysteines. Recent physiochemical investigations and expression profile studies have shown that HFBs and CPs are probably functionally complementary (Gaderer *et al*., 2014). Proteins from both of these families can spontaneously assemble in interfaces, mediate the hydrophobicity of surfaces and reduce water tension (Linder *et al*., 2005; Gaderer *et al*., 2014). These properties attract research attention to HFBs for the development of multiple industrial or pharmacological applications, and to CPs for their ability to trigger plant immune responses and their role in plant pathogenicity.

Fungi from the fungivorous genus *Trichoderma* as well as a few other hypocrealean taxa are considered suitable models for the functional characterization of HFBs and CPs, because the respective gene families are expanded in their genomes (Kubicek *et al*., 2019; Gao *et al*., 2020). In contrast to the majority of Ascomycota fungi, which have a few HFBs and usually only one or two CPs (Chen *et al*., 2013), *Trichoderma* spp. can have seven (e.g. *T*. *reesei* and *T*. *citrinoviride*) to 15 (e.g. *T*. *harzianum* and *T*. *gamsii*) HFB‐encoding genes (Kubicek *et al*., 2019). All whole‐genome sequenced *Trichoderma* strains (September 2020) have at least three CPs, and a few species have four (Gao *et al*., 2020). Moreover, *Trichoderma* spp. are ecologically versatile, and several environmentally opportunistic species can parasitize other fungi, colonize dead wood and other cellulosic substrates, grow in soil, establish in rhizosphere, become endophytes, kill water moulds (Oomycota), control plant‐parasitic nematodes and attack immunocompromised humans (Druzhinina *et al*., 2011). Thus, the expansion of surface‐active SSCPs (saSSCPs) such as the HFBs and CPs in *Trichoderma* genomes may be linked to the diversity of the biotic and abiotic interactions of these fungi.

This study continues the evolutionary and functional characterization of saSSCPs in *Trichoderma* (Kubicek *et al*., 2008; Gao *et al*., 2020). We detected and characterized a new family of saSSCPs, named here as hyphosphere proteins (HFSs) and the two new *Trichoderma* HFBs, here named HFB11 and HFB12. The application of recombinant HFB2, HFB4, HFB12, HFS1 and EPL1 proteins (produced by *Pichia pastoris*) to *Solanum lycopersicum* (tomato) roots resulted in divergent effects on fungal attachment and in a noticeable phytotoxic effect, while the complete secretome of *T*. *guizhouense* stimulated tomato growth. Interestingly, all recombinantly produced saSSCPs (HFS1, HFB2, HF4, HFB12 and EPL1) showed convergent surface‐modulating activity that favoured the attachment of hyphae indicating that the high diversity of saSSCPs in *Trichoderma* likely evolved for the efficient conditioning of various surfaces in the hyphosphere to a hydrophilic state, making it suitable for attachment or colonization by the fungus.

## Results

### Genome mining of *T*. *guizhouense* revealed a new family of SSCPs and two new HFBs

We first performed a broad screening of the whole‐genome sequence of *T*. *guizhouense* NJAU 4742 (Druzhinina *et al*., 2018; Kubicek *et al*., 2019) for saSSCPs such as HFBs and CPs. For this purpose, we expanded the previously available annotations (Kubicek *et al*., 2019) and collected the small (<30 kDa, <300 a.a.) secreted proteins that contained four to eight single or double cysteine residues (see Experimental procedures for further details). The results, shown in Table 1 and in the Supporting Information 1 Table S1, confirmed the three CPs characterized in this species by Gao *et al*. (2020). These data also revealed that there are two putative HFBs described by Kubicek *et al*. (2019) do not belong to this family. The first (GenBank ID: OPB44529) lacks the signal peptide. A comparison with other genomes revealed that it is likely an orphan *Trichoderma* protein that is present in section *Trichoderma* (e.g. *T*. *gamsii*, *T*. *atroviride* and *T*. *asperellum*) and *Harzianum* clade species but absent in the *Longibrachiatum* clade. The second putative HFB (GenBank ID: OPB38365) has the HFB‐specific cysteine pattern of C‐CC‐C‐C‐CC‐C, but it harbours six more cysteines (14 total) and has a size equivalent to that of three HFBs (28 kDa, 277 a.a.). Although it is probably a member of the SSCPs, it presents as a single copy protein that is relatively conserved in all mined ascomycetes, differing from the HFB family. Thus, we concluded that OPB38365 possibly represents a separate family of SSCPs and excluded it from this investigation. We also found two novel putative HFB‐encoding genes that were previously detected by Kubicek *et al*. (2019), which we name here as *hfb11* (OPB42521, encoding HFB11) and *hfb12* (OPB45278, encoding HFB12). Thus, the *T*. *guizhouense* genome encodes at least 10 confirmed HFBs (Table 1). Moreover, we found three other proteins with primary structures fulfilling our search criteria (Table 1). These proteins (OPB41482, OPB43804 and OPB40307) had a pattern of eight single cysteine residues and were larger than the HFBs (7–16 kDa) and CPs (12–14 KDa), with molecular weights varying from 21 to 28 kDa and sequences contained 219–280 residues.

**Table 1:** Inventory and expression of surface‐active SSCPs of *Trichoderma guizhouense*. [Colour table can be viewed at wileyonlinelibrary.com]

saSSCP family	Protein	Protein ID	*M*_w_	SP	Gene	Relative expression, folds					
						Submerged growth		Aerial hyphae		Conidiation	
			Da	a.a.		Av.	*Sd*	Av.	*Sd*	Av.	*Sd*
Hydrophobins (HFBs)	HFB2	OPB38530	7044.17	16	*hfb2*	**0.083821**	*0*.*032*	**0.407867**	*0*.*110*	**0.008979**	*0*.*004*
	HFB3	OPB45549	8923.33	16	*hfb3*	**0.004052**	*0*.*002*	**0.168363**	*0*.*042*	**0.007011**	*0*.*005*
	HFB4	OPB37525	8486.68	21	*hfb4*	**0.004519**	*0*.*001*	**0.641103**	*0*.*272*	**0.335277**	*0*.*262*
	HFB5	MF527119	8282.49	16	*hfb5*	**0.000049**	*0*.*000*	**0.002428**	*0*.*001*	**0.015500**	*0*.*014*
	HFB6	OPB38878	15540.76	16	*hfb6*	**0.000000**	*0*.*000*	**0.000000**	*0*.*000*	**0.000000**	*0*.*000*
	HFB9a	OPB40515	14414.52	18	*hfb9a*	**0.000003**	*0*.*000*	**0.000004**	*0*.*000*	**0.000149**	*0*.*000*
	HFB9b	OPB44528	12117.36	18	*hfb9b*	**0.001415**	*0*.*000*	**0.003648**	*0*.*001*	**0.001090**	*0*.*001*
	HFB10	OPB44696	11649.88	16	*hfb10*	**5.321598**	*2*.*544*	**7.377791**	*2*.*857*	**1.071798**	*0*.*843*
	HFB11	OPB42521	7590.57	20	*hfb11*	**0.000000**	*0*.*000*	**0.000000**	*0*.*000*	**0.000000**	*0*.*000*
	HFB12	OPB45278	7571.42	16	*hfb12*	**0.000002**	*0*.*000*	**0.000010**	*0*.*000*	**0.000668**	*0*.*000*
Cerato‐platanins (CPs)	EPL1	OPB44018	12461.81	18	*epl1*	**0.380258**	*0*.*139*	**0.104417**	*0*.*029*	**0.012128**	*0*.*008*
	EPL2	OPB45524	13169.57	18	*epl2*	**0.000005**	*0*.*000*	**0.001414**	*0*.*000*	**0.003207**	*0*.*002*
	EPL3	OPB43811	14277.99	18	*epl3*	**0.000023**	*0*.*000*	**0.000023**	*0*.*000*	**0.000113**	*0*.*000*
Hyphosphere proteins (HFSs)	HFS1	OPB41482	21856.39	20	*hfs1*	**0.000043**	*0*.*000*	**0.000053**	*0*.*000*	**0.000085**	*0*.*000*
	HFS2	OPB43804	21271.61	18	*hfs2*	**0.000133**	*0*.*000*	**0.000185**	*0*.*000*	**0.000092**	*0*.*000*
	HFS3	OPB40307	27841.06	20	*hfs3*	**0.017148**	*0*.*002*	**0.037480**	*0*.*007*	**0.025919**	*0*.*002*

*M*_w_, molecular weight; SP, signal peptide; Av., average; *Sd*, standard deviation from four repeats; a.a., amino acid.

**Fig. 1:**
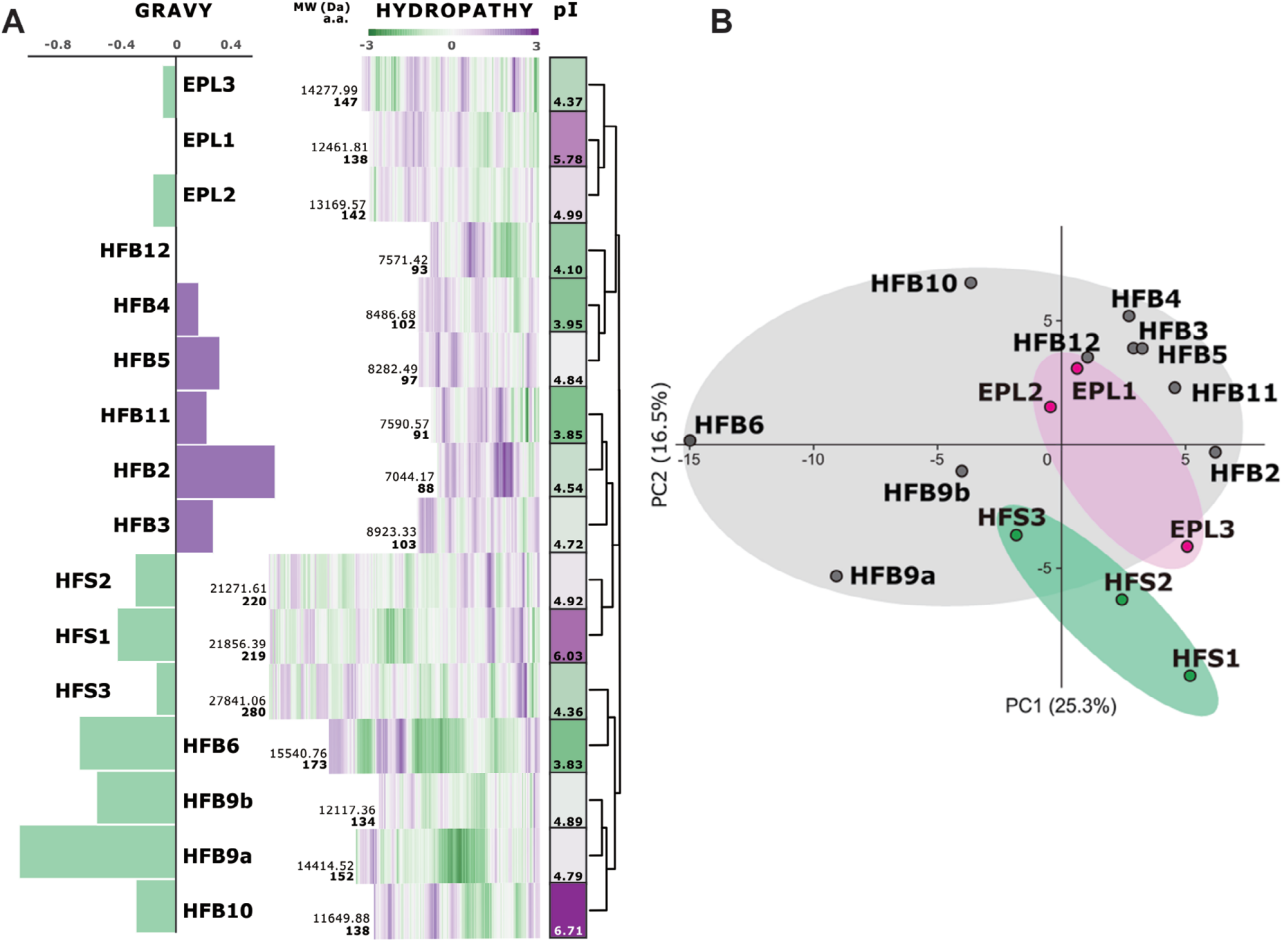
Primary structure analysis of surface‐active small secreted cysteine‐rich proteins (SSCPs) in *Trichoderma guizhouense*. A. Deep purple colour highlights relatively high values of either the hydrophobicity based on protein GRAVY scores, the Kyte and Doolittle hydrophobicity coefficient (Kyte and Doolittle, 1982) for individual residues, or the isoelectric point (pI). Green corresponds to the respective low values. The cluster analysis on the right was applied to the hydropathy profile values using complete linkage and Euclidian distances. B. Principal component analysis (PCA) of the Kyte and Doolittle hydropathy coefficients, which shows a high similarity between proteins from the three families (i.e. both components explain minor variability).

As the physiochemical surface‐active properties of the HFBs and CPs in *Trichoderma* spp. are relatively well studied (Kubicek *et al*., 2008; Seidl‐Seiboth *et al*., 2011; Espino‐Rammer *et al*., 2013; Bonazza *et al*., 2015; Przylucka *et al*., 2017b; Gao *et al*., 2020), we compared their hydropathy profiles to those of the newly detected proteins (Fig. 1A). The principal component analysis (PCA) based on the Kyte and Doolittle hydrophobicity coefficient (Kyte and Doolittle, 1982) and the values of the GRAVY scores, which define a protein sequence's average degree of hydrophobicity, showed that the novel proteins had hydropathy profiles similar to those of the HFBs and CPs (Fig. 1B) and therefore had the potential for being surface‐active or amphiphilic. In particular, the GRAVY scores suggested these novel proteins were comparable to HFB6 and the pseudo‐class I HFB members HFB9a/b in *Trichoderma* (Seidl‐Seiboth *et al*., 2011). As they have a unique pattern of eight single cysteine residues (see below) and are considerably larger in size, we assigned them to a putative new family of saSSCPs and named them HFSs what is phonetically similar to ‘hyphosphere’ proteins, see below: HFS1 (OPB41482), HFS2 (OPB43804) and HFS3 (OPB40307).

In general, the primary structure analysis indicated that HFB2, HFB3, HFB4, HFB5 and HFB11 are potentially more hydrophobic than HFB6, HFB9a, HFB9b, HFB10, HFS1 and HFS2, which are somewhat hydrophilic. Proteins such as HFB12, CPs (EPL1, EPL2 and EPL3) and HFS3 have intermediate values. The isoelectric point (pI) analysis showed no correlation with a particular family or hydropathy profile, with ranges varying from 3.83 to 6.71 in the HFBs, 4.37 to 5.78 in the CPs and 4.36 to 6.03 in the HFSs. Only a few proteins, namely EPL1, HFS1 and HFB10, had pIs close to the 6–7 range, while the majority of the others could be considered ‘cationic’ (Huber *et al*., 2020), suggesting they would require unique environmental conditions and potentially precipitate under neutral (pH 7; like water) conditions. All 16 proteins have a relatively short signal peptide (16–21 a.a.), which is characteristic of HFBs (Kottmeier *et al*., 2011).

To confirm the functionality of the saSSCP‐encoding genes, we tested their expression during the main stages of the asexual life cycle of *T*. *guizhouense* when these proteins are known to be active. This included submerged vegetative growth that associated with high CP expression (Gaderer *et al*., 2014; Gao *et al*., 2020), and aerial hyphae formation and conidiation which require large amounts of HFBs (Lugones *et al*., 2004; Winefield *et al*., 2007; Cai *et al*., 2020). The qPCR analysis confirmed the expression of all CP‐ and HFB‐encoding genes except *hfb6* and *hfb11* (Table 1). The expression of *hfb12* was low compared with major *hfb*s such as *hfb4*, *hfb10*, *hfb2* and *hfb3* (Cai *et al*., 2020), but showed the *hfb*‐characteristic pattern of a drastic (several hundred folds) increase with the formation of aerial hyphae and conidiation. The three genes encoding HFS proteins showed a relatively low but consistent expression profile that did not change much with the development of the fungus (Table 1). Thus, our data suggest that at least 14 of the detected putative saSSCP‐encoding genes are actively transcribed, while the other two could either be putative pseudogenes or become functional under specific conditions not tested here.

### Evolutionary analysis suggests a long evolutionary history of HFSs in filamentous Ascomycota

**Fig. 2:**
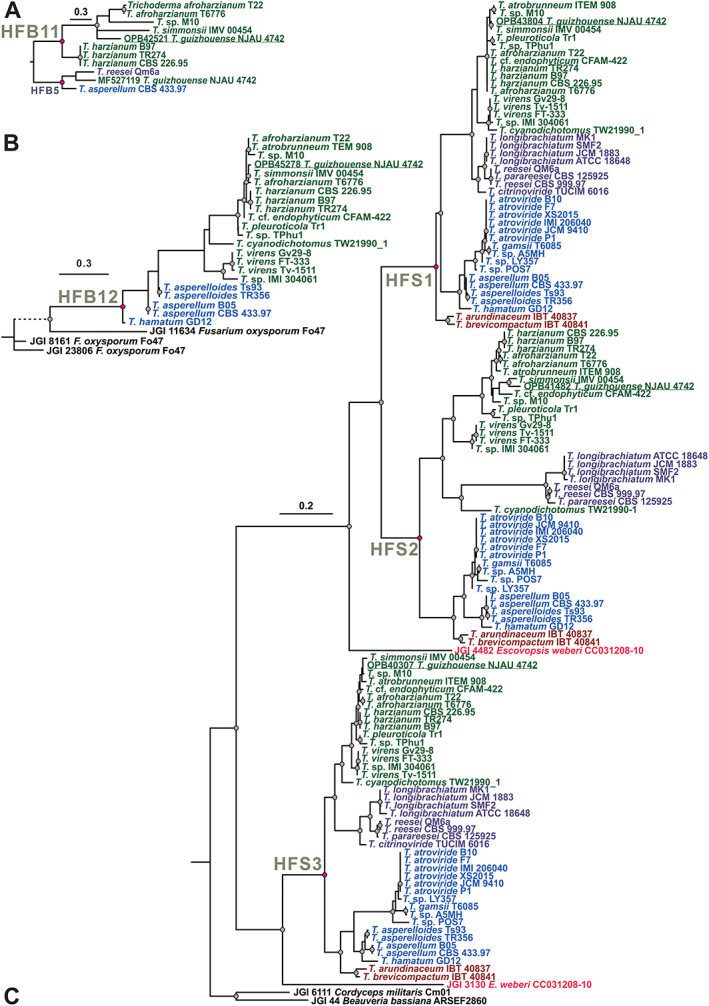
Phylogenetic analysis of newly described surface‐active small secreted cysteine‐rich proteins (saSSCPs) from *Trichoderma* spp. The evolution of HFB11 (A), HFB12 (B) and HFSs (C) was inferred based on the amino acid alignments. Maximum‐likelihood (ML) phylogenetic trees were constructed using IQ‐TREE 1.6.12 (ultrafast bootstrap, *N* = 1000). The Bayesian Information Criterion was applied when searching for the best amino acid substitution model with ModelFinder (integrated into the IQ‐TREE program). Statistically supported nodes (bootstrap values >75) are marked by dots. Pink dots indicate the hypothetical ancestor for the particular protein. *Trichoderma* spp. from the *Harzianum* and *Virens* clades, section *Longibrachiatum*, section *Trichoderma*, the *Brevicompactum* clade and *Escovopsis weberi* are highlighted in dark green, purple, blue, orange and red, respectively, for improved visual assessment. The accession numbers of the individual genomes mined in this study are provided in Supporting Information 1 Table S2.

The newly described HFB11 in *T*. *guizhouense* is 91 a.a. long and has a molecular weight of 7.59 kDa. This protein also has the characteristic C‐CC‐C‐C‐CC‐C pattern and a short signal peptide (20 a.a.). The evolutionary analysis revealed it to be an orphan protein present in only a few species in the *Harzianum* clade of the genus *Trichoderma* (Fig. 2A), including *T*. *simmonsii*, *T*. *afroharzianum*, *T*. *harzianum* and the putatively new species *T*. sp. M10 (Cai and Druzhinina, 2021). We did not detect any *hfb11* sequences in *Harzianum* clade species such as *T*. *pleuroti* and *T*. *pleuroticola* or in the sister clade *Virens*. The amino acid sequence of HFB11 was ~50% similar to HFB5 (*hfb5*, MF527119; Fig. 2A), which is in agreement with the similar primary structure of these two proteins (Fig. 1).

Another newly described HFB12 has a sequence of 93 a.a. harbouring the typical C‐CC‐C‐C‐CC‐C cysteine pattern of HFBs that provides for an *M*_w_ of 7.57 kDa with a 16 a.a. signal peptide. The phylogenetic analysis (Fig. 2B) showed that among the 42 *Trichoderma* spp. (August 2020) whole‐genome sequences, *hfb12* can be found in the *Harzianum* and *Virens* clades, *T*. *cyanodichotomus* (which is phylogenetically close to *T*. *virens*), and in some species of section *Trichoderma* such as *T*. *asperellum*, *T*. *asperelloides* and *T*. *hamatum*, but not in the species *T*. *atroviride* or *T*. *gamsii*. The gene is also lacking in the *Brevicompactum* clade and section *Longibrachiatum*. Interestingly, the phylogeny of HFB12 was not concordant with the phylogeny of the genus (Kubicek *et al*., 2019), which indicated the likely scenario of a gene loss (GL) event in section *Longibrachiatum* and a partial loss in the other clades. No homologues to the *hfb12* genes were found in other fungi, though the closest neighbour was <50% similar to the *Fusarium oxysporum* species complex (Hypocreales, Ascomycota). Phylogenetically, *hfb12* was highly divergent compared with the other HFBs in *Trichoderma*. Thus, we assume *hfb12* is an orphan *Trichoderma* gene that is not present in the core genome of the genus or in other fungi.

To investigate the evolution of the *T*. *guizhouense* HFSs, we performed a sequence similarity search (BLASTP) against the NCBI and JGI databases and screened all 42 publicly available *Trichoderma* genomes [August 2020; (Cai and Druzhinina, 2021)] as well as the genome of the closely related hypocrealean fungus *Escovopsis weberi* (de Man *et al*., 2016). The multiple sequence alignment revealed that the pattern of eight single cysteine residues of HFSs was highly conserved in all *Trichoderma* spp. For example, the HFS2 in *T*. *reesei* (XP_006967391) had a structure of C_49_‐C_65_N_66_N_67_L_68_C_69_‐C_129_‐C_152_‐C_174_‐C_182_‐C_194_. In other fungi (see below), the amino acids and length of the sequence between the C residues slightly varied, except for the space between the second and third cysteines, which were always separated by three amino acids; the first two were most frequently asparagine (N) or aspartic acid (D), while the third was an aliphatic amino acid such as leucine (L) or isoleucine (I). The maximum likelihood (ML) phylogram in Fig. 2C demonstrated that the three HFS proteins were homologous and shared the statistically supported common ancestor. The ancestor of HFS1 and HFS2 was, in turn, monophyletic with HFS3. In *Trichoderma*, the similarity between the amino acid sequences of the proteins varied from 62% to 100% for HFS1, 78% to 100% for HFS2 and 71% to 100% for HFS3. Three HFS‐encoding genes were harboured in all the screened *Trichoderma* genomes with the exception of *T*. *citrinoviride*, which was missing HFS2. In general, the topologies of the individual HFS subclades were concordant with the topology of the *Trichoderma* phylogenomic tree (Kubicek *et al*., 2019), suggesting the vertical evolution and stabilizing selection pressure (Druzhinina *et al*., 2018; Cai *et al*., 2020; Gao *et al*., 2020) that were recorded for the other groups of secreted proteins in *Trichoderma*.

**Fig. 3:**
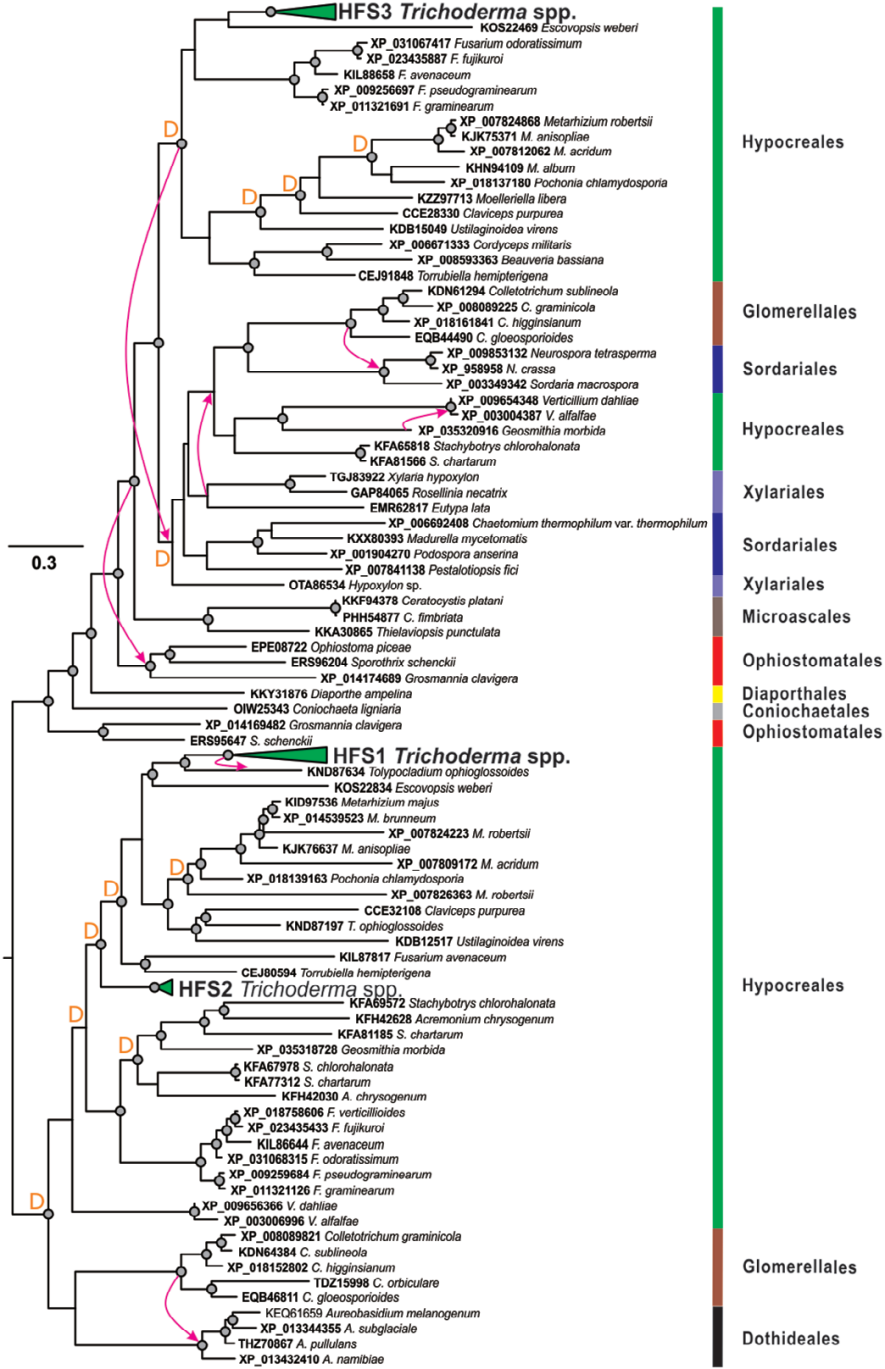
Evolution of the hyphosphere (HFS) proteins in fungi. A maximum‐likelihood phylogenetic tree was constructed using IQ‐TREE 1.6.12 (ultrafast bootstrap, *N* = 1000). The Bayesian Information Criterion was applied when searching for the best amino acid substitution model with ModelFinder (integrated into the IQ‐TREE program). Statistically supported nodes (bootstrap values >75) are marked by dots. Arrows indicate putative cases of lateral gene transfer as revealed by NOTUNG and T‐Rex; ‘D’ indicates the putative gene duplication events revealed through the same methods. Vertical bars denote the taxonomic positions of the corresponding species.

The topology of the HFS phylogram suggests a GD event in the ancestor of the genus *Trichoderma* that resulted in paralogous copies of *hfs1* and *hfs2*. To test this, we collected HFS sequences from other fungi and performed NOTUNG and T‐Rex analyses (Chen *et al*., 2000; Boc *et al*., 2012; Gao *et al*., 2020), which allowed us to test for possible GD, GL and LGT events in the evolutionary history of the HFSs in fungi. The results showed that HFSs are common in filamentous Ascomycota, but absent in other Dikarya fungi. The analyses confirmed the evolution of HFS1 and HFS2 by GD (Fig. 3), showing that the event most likely took place in the ancestor of hypocrealean fungi. One duplicated copy of *hfs1* was inherited by the fungi from most other families, while *hfs2* was only maintained in *Trichoderma* and likely lost in the other families. Interestingly, our analysis showed that *hfs1* was putatively laterally transferred to the genome of *Tolypocladium ophioglossoides* (Hypocreales, Ascomycota), which has its own copy of *hfs1* that was obtained vertically. The third gene, *hfs3*, also evolved as a result of a GD event but only one copy was maintained in *Trichoderma*. Similar to the evolution of CPs (Gao *et al*., 2020), the evolutionary history of the HFSs in filamentous Ascomycota encountered numerous cases of LGTs, GDs and GLs, all indicating the functional significance of these proteins. Our data show that the genes encoding HFSs were present in parasites feeding on any type of live biomass and in polyphages feeding saprotrophically on different organic matter (Fig. 3). Thus, the evolutionary analysis indicates the functional importance of these genes for filamentous Ascomycota, but it does not suggest that the acquisition of these genes was associated with a particular nutritional strategy or lifestyle of the fungi.

### Heterologous production of saSSCPs in *P*. *pastoris*

**Fig. 4:**
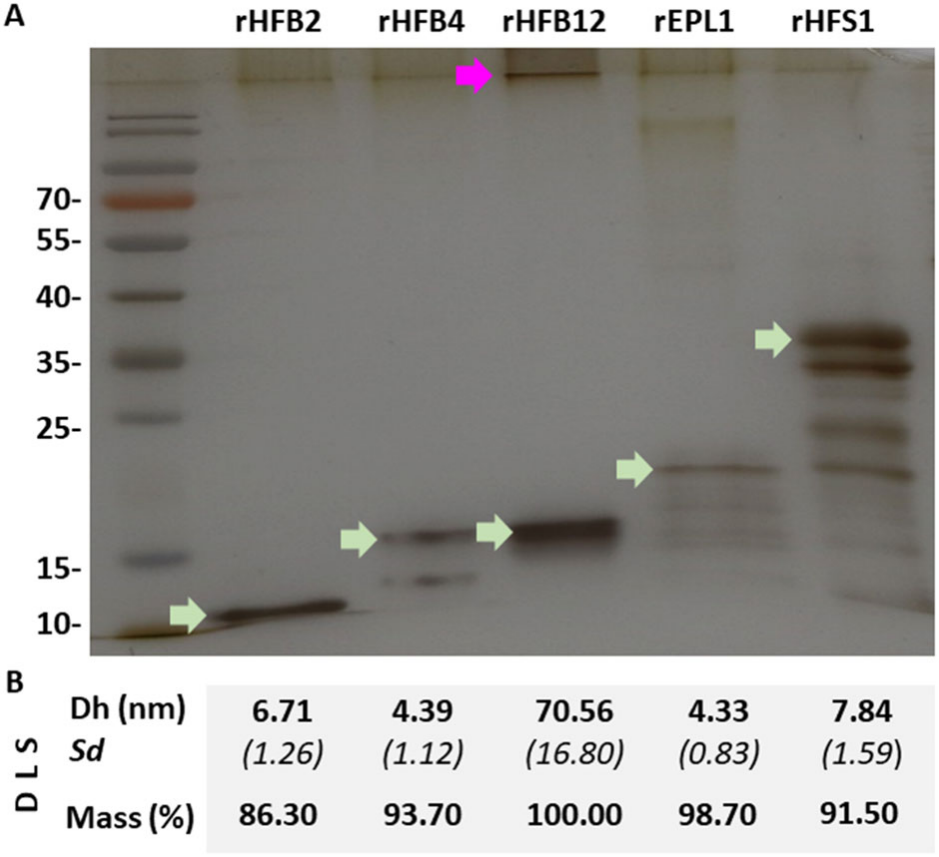
Recombinant production of the five surface‐active small secreted cysteine‐rich proteins (saSSCPs) from *Trichoderma guizhouense* in *Pichia pastoris*. A. The SDS‐PAGE analysis of recombinant saSSCPs (green arrows). The migration of HFB12 through the polyacrylamide gel was impeded (purple arrow). B. Results of the dynamic light scattering analysis (DLS) applied to the same samples shown in A. Dh corresponds to the mass weight (%) diameter of the proteins obtained from three measurements. *Sd*, standard deviation.

In previous studies, we explored the use of a gene deletion strategy for the functional characterization of saSSCPs in *Trichoderma* (Cai *et al*., 2020; Gao *et al*., 2020). However, the high number of SSCPs and the low expression profile of the newly revealed proteins indicated that this approach would not be suitable for their characterization. Therefore, we selected HFB12 and HFS1 for recombinant production in a *Komagataella phaffii* [syn. *Pichia pastoris* (Kurtzman, 2009)] host. For the comparison, we used the previously characterized saSSCPs of *Trichoderma* such as the HFBs [HFB2, (Askolin *et al*., 2005), HFB4 (Cai *et al*., 2020)] and a CP [EPL1; (Bonazza *et al*., 2015; Gao *et al*., 2020)]. For the construction of mutants in *P*. *pastoris*, the production of each protein was confirmed by SDS‐PAGE (Fig. 4A) and immunoblotting (Supporting Information 2 Fig. S1). The flask fermentation of the engineered *P*. *pastoris* strains yielded at least 0.3–0.5 g L^−1^ of recombinant protein, varying slightly between the proteins produced and the fermentation batches. The expected size of each recombinant protein, shown in the SDS‐PAGE, excluded the possibility of glycosylation in the recombinants.

The behaviour of each recombinant protein (indicated by ‘r’) in water was estimated using dynamic light scattering (DLS) (Przylucka *et al*., 2017b). The hydrodynamic diameters of the proteins based on the mass weight (%) are shown in Fig. 4B. The majority (mass > 90%) of the rHFB2, rHFB4 and rEPL1 proteins displayed a hydrodynamic size of 4.3–6.7 nm, which corresponded to that of a monomer or dimer of previously reported proteins (Hakanpää *et al*., 2004; Przylucka *et al*., 2017b). The rHFS1 protein had a slightly larger particle size of ~7.8 nm. Interestingly, rHFB12 was found mainly (100%) in large aggregates with a dynamic size >70 nm, which could also be seen in the intensity distribution plot (Supporting Information 2 Fig. S2). Therefore, using HFB1 [2–3 nm; (Hakanpää *et al*., 2004)] from *T*. *reesei* as a reference for monomers, rHFB2, rHFB4, rEPL1 and rHFS1 tended to form dimers or oligomers in a water solution, while rHFB12 easily formed aggregates, explaining the retention of this protein in the sample well during the SDS‐PAGE analysis (Fig. 4A).

### All saSSCPs convert surfaces to a moderately hydrophilic state

**Fig. 5:**
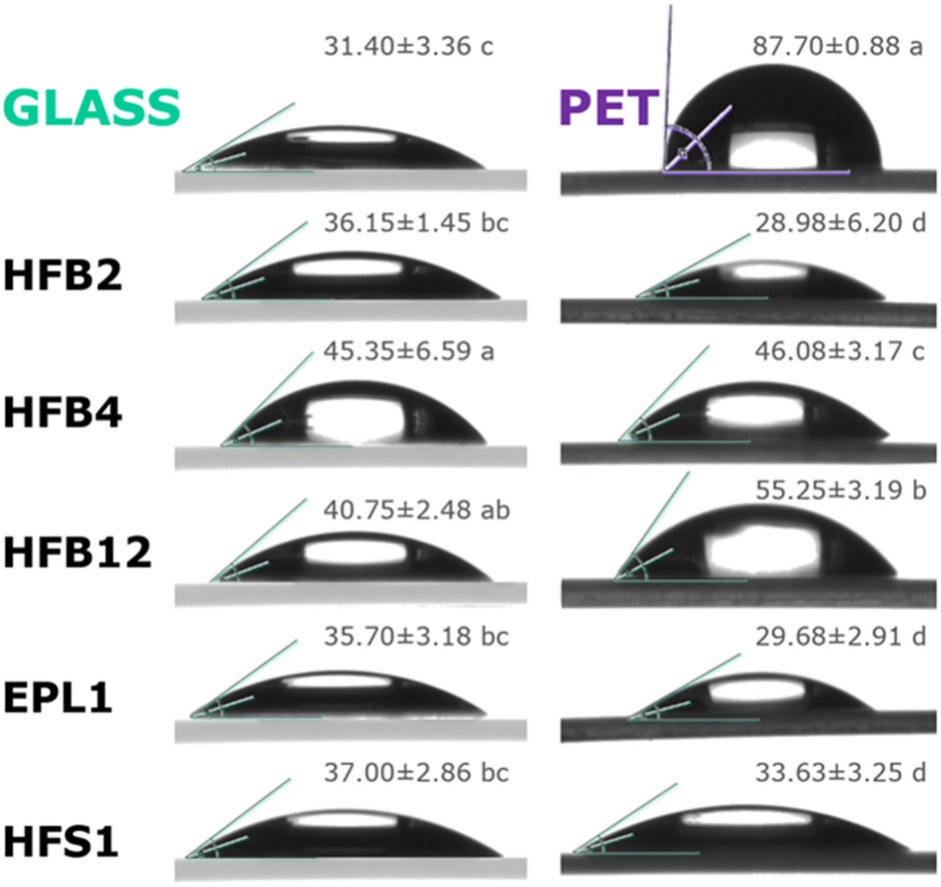
Surface‐modulating activity of the recombinant surface‐active small secreted cysteine‐rich proteins (rsaSSCPs) from *Trichoderma guizhouense* revealed by a water contact angle analysis (WCA) on glass and poly(ethylene terephthalate) (PET). Values (mean ± *Sd*) labelled with the same letter do not statistically significantly differ (ANOVA, *p* < 0.05).

**Fig. 6:**
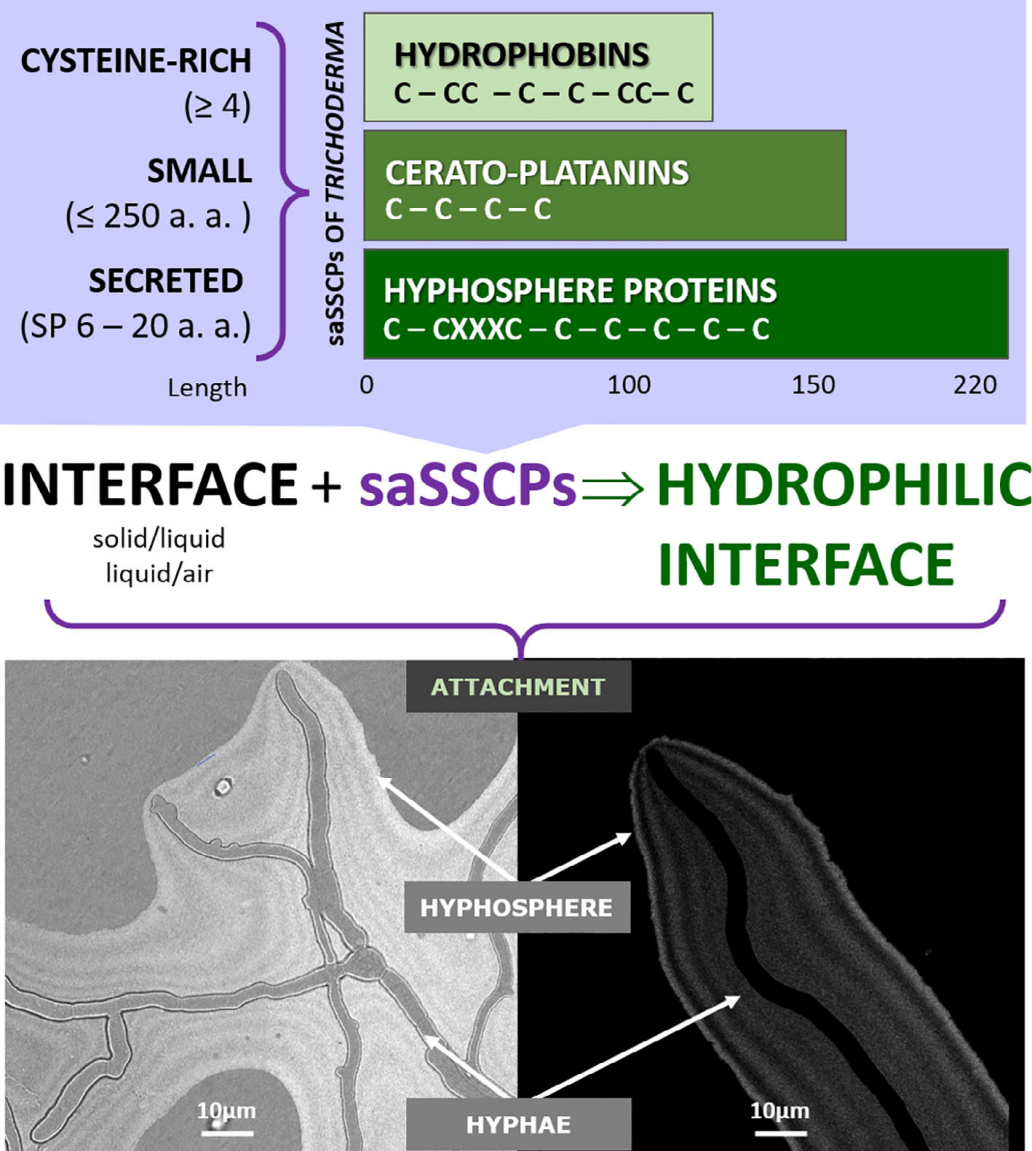
Diagram showing the main distinctive features of the three families of surface‐active small secreted cysteine‐rich proteins (saSSCPs) in *Trichoderma guizhouense* and their hypothetical function in the hyphosphere. The young hyphae of *T*. *guizhouense* NJAU 4742 were imaged on a cellophane surface covering a potato dextrose agar plate 18 h after inoculation (incubated at 25 °C in the dark). The stripes in the hyphosphere correspond to unequal light reflection, likely due to the assembly of different saSSCPs. The right and left images depict a hyphosphere of multiple (left) and a single (right) hyphae imaged using similar setups.

The measurements of the water contact angle (WCA) on initially hydrophobic [poly(ethylene‐terephthalate), PET] and hydrophilic (glass) surfaces coated with the recombinant saSSCPs showed that all five proteins were surface‐active and provided a remarkably consistent result (Fig. 5). Irrespective of the initial hydrophobicity of the material, all proteins converted both surfaces to a moderately hydrophilic state, which corresponds to a WCA between 30° and 45°, excluding rHFB12 on PET. Thus, rHFB4 and rHFB12 increased the surface hydrophobicity of glass (30%–44%, *p* < 0.05) and decreased that of PET (37%–51%, *p* < 0.05). The rHFB2, rEPL1 and rHFS1 coatings on glass surfaces did not differ from the control, but the hydrophobicity of PET was reduced by 70% (*p* < 0.05). As predicted by the primary structure analysis, the newly detected SSCPs (HFS1 and HFB12) have potent surface activity similar to that of HFBs and CPs. The aggregation of HFB12 in water (see above) did not abolish its surface activity, although it was slightly reduced compared with that of the other tested saSSCPs. Considering the shared evolutionary history of the three HFSs and the similarity of their hydropathy profiles, they can be assigned as a family of saSSCPs (Fig. 6). More interestingly, the results indicate that *T*. *guizhouense* secretes an arsenal of saSSCPs that are all capable of transforming the surfaces surrounding hyphae to a moderately hydrophilic state. The consistency of the results allows us to hypothesize that hydrophilic surfaces corresponding to a 30°–45° WCA are optimal for the fungus.

### saSSCPs assist *Trichoderma* hyphal attachment to hydrophilic surfaces

**Fig. 7:**
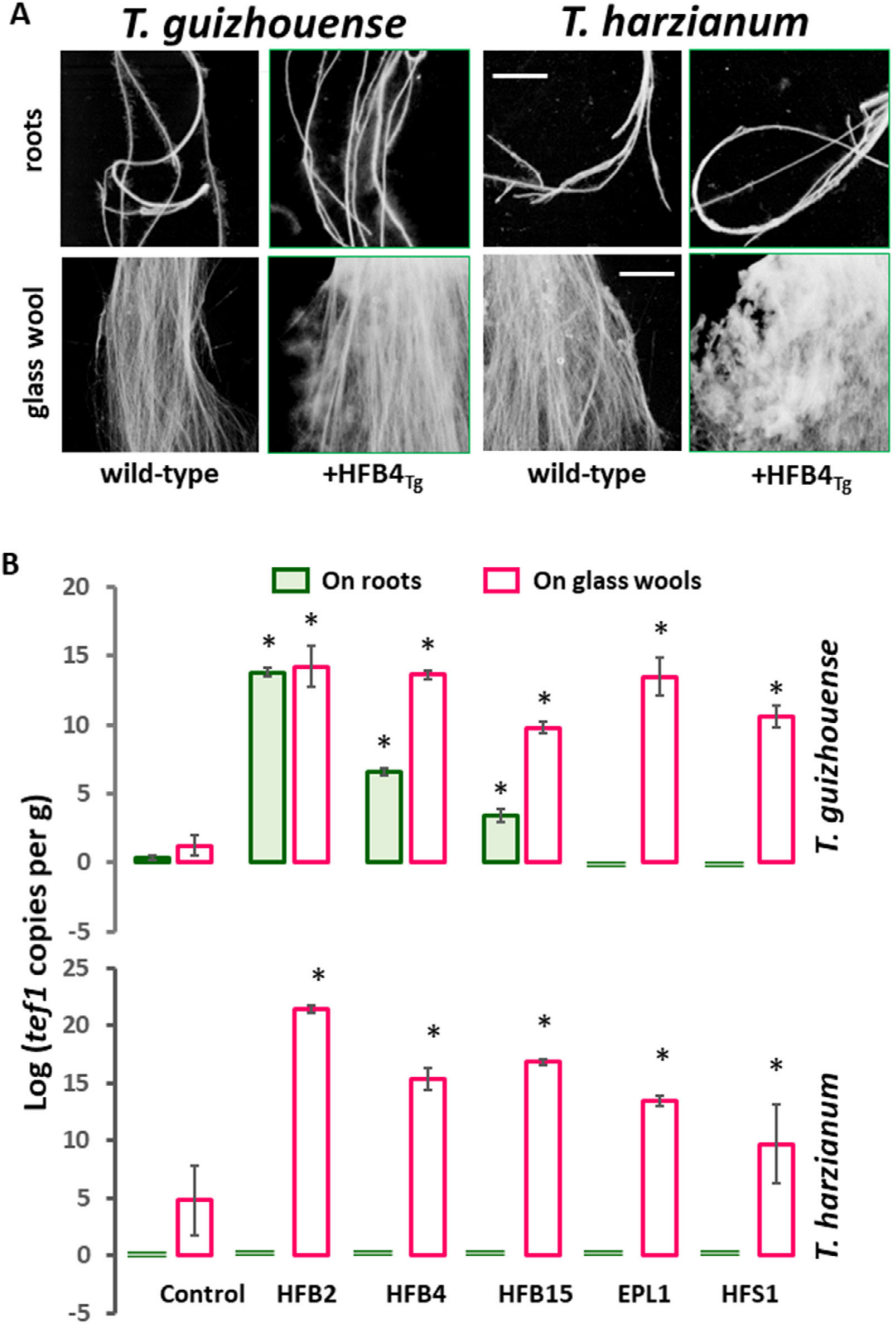
Effect of recombinantly produced surface‐active small secreted cysteine‐rich proteins (saSSCPs) on the ability of *Trichoderma* hyphae to attach to biotic (tomato roots) and abiotic (glass wool) surfaces. A. Sample images obtained for *T*. *guizhouense* and *T*. *harzianum* with or without addition of recombinantly produced HFB4. Images were obtained using a root scanner after 48 h of incubation. Scale, 1 cm. B. Quantification of fungal attachment to biotic (tomato roots) and abiotic (glass wool) surfaces using a qPCR determination of the single copy constitutively expressed house‐keeping gene *tef1* (Cai *et al*., 2020; Gao *et al*., 2020).

As *T*. *guizhouense* has the ecophysiological features of an aero‐aquatic fungus (Cai *et al*., 2020) and is also rhizosphere competent (Cai *et al*., 2013, 2015; Zhang *et al*., 2016), we hypothesized that saSSCPs that are frequently expressed during submerged growth (Table 1) and transform surfaces to a moderately hydrophilic state (similar to that of glass) could also assist mycelial attachment to such modified hydrophilic surfaces in water. (Note that due to the abundance of saSSCPs, all hydrophobic surfaces would ultimately become hydrophilic.) To model such surfaces, we used glass wool (artificial) and the roots of *Solanum lycopersicum* (tomato) seedlings (natural substrate). The results showed that the *T*. *guizhouense* strain had a higher affinity to the glass wool, while attachment to roots was less successful (Fig. 7). The addition of 1 μM of any rsaSSCPs produced a significant (*p* < 0.05) and multifold increase in hyphae attachment to the glass wool. As for the attachment to roots, three rHFBs produced a similar result, improving colonization, while rEPL1 and rHFS1 had a reverse impact, preventing hyphae attachment. To see whether these functions were species specific, we performed the same experiment with another *Trichoderma* strain, *T*. *harzianum* CBS 226.95, which is related to *T*. *guizhouense* (Kubicek *et al*., 2019) but has a different ecologic profile (Cai *et al*., 2020). The results for *T*. *harzianum* repeatedly showed that rsaSSCPs can assist in the attachment of the fungus to glass wool, but none of the selected rsaSSCPs from *T*. *guizhouense* NJAU 4742 could prime the colonization of *T*. *harzianum* CBS 226.95 on tomato roots (Fig. 7). This indicates that root attachment is a complex process that is probably regulated by both partners, and the addition of saSSCPs does not necessarily have an impact.

### Some saSSCPs are toxic for plants and can trigger an immune response

**Fig. 8:**
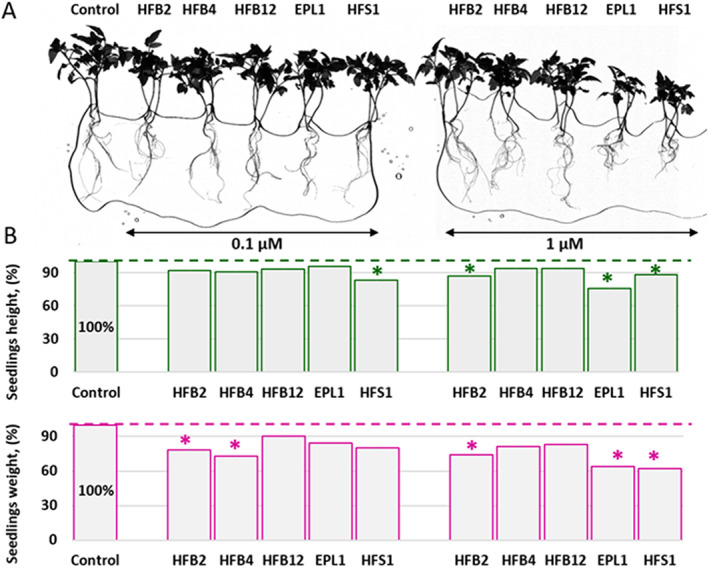
Phytotoxic effect of recombinant surface‐active small secreted cysteine‐rich proteins (rsaSSCPS) on tomato growth. A. Tomato plants imaged using a root scanner after 21 days of hydroponic growth with or without the addition of two concentrations of rsaSSCPS. B. Quantification of tomato growth based on seedling height and weight. Values that significantly differ from the control are marked by asterisks (ANOVA, *p* < 0.05). See A for the concentrations of the rsaSSCPs.

As *T*. *guizhouense* itself and its secretomes have considerable growth‐promoting effects on plants [Supporting Information 2 Fig. S3 and Cai *et al*., 2013, Cai *et al*., 2015], we tested the effect of the rsaSSCPs on the growth of tomato seedlings using 0.1 and 1 μM of a purified protein suspension. Surprisingly, with the exception of HFB12, these treatments had a significantly adverse effect on plant growth that varied in strength and range between the different rsaSSCPs (Fig. 8). One micromole of rHFS1 and rEPL1 significantly (*p* < 0.05) reduced plant growth to 62% and 64% respectively, of the control biomass without the protein addition. The rHFB2 and rHFB4 proteins had a less severe effect (73%–78%) on plant growth, though it was still statistically significant (*p* < 0.05) when compared with the control, and the plant height was reduced accordingly. The rHFB12 protein had a neutral effect on the plants, which may be due to its strong aggregation in water (Fig. 4). The growth inhibition effects of the protein application increased when the concentration was raised from 0.1 to 1 μM (Fig. 8).

Since saSSCPs are frequently considered effector‐like proteins (Gaderer *et al*., 2014; Ruocco *et al*., 2015; Gao *et al*., 2020), we also tested whether the immune system of the tomato seedlings was activated by the addition of the rsaSSCPs. To this end, we monitored the expression of biomarker genes for the jasmonic acid (JA)‐ and salicylic acid (SA)‐defence pathways in plants. The results (Table 2) show that 0.1 μM of rHFB2 and rHFB12 slightly but significantly (*p* < 0.05) activated the JA‐ and SA‐mediated defence pathways, as shown by the upregulation of the respective genes *LoxA* (11–13‐fold) and *PAL* (~two folds). The rEPL1 protein did likewise. However, the elicitation effect of these proteins was not seen when the protein concentration was increased to 1 μM. In contrast, neither rHFB4 nor rHFS1 was able to trigger the plant systemic defence pathways at the two applied concentrations, likely because under natural conditions, HFB4 is associated with conidiation (Cai *et al*., 2020) and thus secreted in very low amounts in the rhizosphere, and the expression of HFS1‐encoding gene is generally low (Table 1).

**Table 2:** Relative expression of *Solanum lycopersicum* (tomato) genes involved in induced systemic resistance after application of rsaSSCPs to roots.

Tomato genes	*AOS*				*LoxA*				*PAL*				*PR‐1a*			
rsaSSCP conc. (μM)	0.1		1.0		0.1		1.0		0.1		1.0		0.1		1.0	
	Av.	*Sd*	Av.	*Sd*	Av.	*Sd*	Av.	*Sd*	Av.	*Sd*	Av.	*Sd*	Av.	*Sd*	Av.	*Sd*
HFB2	**1.93**	*0*.*38*	**1.17**	*0*.*39*	13.18^*^	*3*.*74*	**3.12**	*0*.*67*	2.54^*^	*0*.*70*	**1.42**	*0*.*20*	**0.71**	*0*.*15*	**0.49**	*0*.*1*
HFB4	**1.37**	*0*.*66*	**0.96**	*0*.*73*	**5.37**	*2*.*25*	**5.51**	*3*.*17*	**1.76**	*0*.*54*	**0.86**	*0*.*59*	**0.29**	*0*.*11*	**0.38**	*0*.*27*
HFB12	**1.34**	*0*.*28*	**1.73**	*0*.*50*	11.26^*^	*3*.*09*	**4.92**	*1*.*30*	**2.21**	*0*.*43*	**1.93**	*0*.*23*	**0.50**	*0*.*06*	**0.45**	*0*.*15*
EPL1	**1.52**	*0*.*54*	**2.29**	*0*.*54*	6.39^*^	*1*.*16*	**3.75**	*1*.*48*	2.62^*^	*0*.*74*	**1.95**	*0*.*18*	**0.47**	*0*.*07*	**0.47**	*0*.*16*
HFS1	**0.93**	*0*.*37*	**2.01**	*0*.*79*	**3.98**	*2*.*46*	**4.03**	*0*.*84*	**1.37**	*0*.*34*	**1.76**	*0*.*71*	**1.08**	*0*.*36*	**1.43**	*0*.*81*

Av., average; *Sd*, standard deviation from four repeats; Statistically significantly upregulated values compared with no rsaSSCP treatment (control) are marked by asterisks (ANOVA, *p* <0.05).

## Discussion

Studies on HFBs and CPs have frequently focused on the effect of these proteins on other organisms. It is not a surprise that plants and animals recognize these massively produced proteins as indicators of fungal proximity, and they respond by activating their defence mechanisms (Frías *et al*., 2011; Wang *et al*., 2019; Gao *et al*., 2020). In plant‐pathogenic fungi, numerous HFBs and CPs have been reported as effector‐like proteins or elicitors (Rohe *et al*., 1995; Whiteford *et al*., 2004; Jeong *et al*., 2007; Frías *et al*., 2011). However, our evolutionary analyses and the study of Gao *et al*. (2020) suggest that their role in plant virulence is likely a secondary function of the saSSCPs, because genes encoding HFBs and CPs are also maintained in the genomes of saprotrophic and animal‐associated fungi.

Besides the detection of a new family of saSSCPs (see below), the results of this study allow us to offer one possible explanation to the overall diversity of HFB‐ and CP‐encoding genes in the genomes of fungi with different lifestyles. We propose that the critical property of HFBs, CPs, the newly detected HFSs and probably other still undetected SSCPs is their ability to modify the interfaces around hyphae (i.e. in hyphosphere). We show that all *T*. *guizhouense* saSSCPs can convert the tested abiotic surfaces to a moderately hydrophilic state and facilitate hyphae attachment to these surfaces. We propose that *T*. *guizhouense* and a dozen other environmentally opportunistic *Trichoderma* spp. have a rich reserve of various surface‐active proteins that can secure their attachment to virtually any surface, thus contributing to the nutritional and ecological versatilities of these fungi. This study showed that several HFBs, CPs and HFSs had very similar surface‐modulating abilities. They all transformed the highly hydrophobic surface of PET (which can resemble a plant or insect cuticle) and hydrophilic glass to the same moderately hydrophilic state. However, the 16 tested genes encoding saSSCPs from different families had unequal expression and regulation profiles. This brings us to the hypothesis that for fungi that perform most of their life‐sustaining functions directly through their body surfaces, suitable interface hydrophobicity in hyphosphere is a critical issue. The regulation of saSSCPs is likely fine‐tuned to particular environmental conditions around the hypha, making its surface and the surfaces around it sufficiently hydrophilic for nutrient absorption or coverage in a hydrophobic ‘raincoat’ to protect it from stressors. This hypothesis can be tested in future studies.

This study showed that all saSSCPs improved the attachment of *Trichoderma* hyphae to a hydrophilic glass surface. Due to the fact that fungi always secrete a diversity of saSSCPs, tests with initially hydrophobic surfaces would not be feasible, as all such surfaces are converted to a hydrophilic state by the activity of such proteins. However, attachment to roots appeared to be a more complicated process. The recombinant HFBs facilitated the attachment of *T*. *guizhouense* to tomato roots, while EPL1 and HFS1 prevented attachment. With or without saSSCPs, *T*. *harzianum* could not attach to tomato roots. Similar results of improved colonization were obtained when the CP *epl1* gene was deleted from *T*. *guizhouense* (Gao *et al*., 2020).

Interestingly, we found several other reports of improved root colonization by fungal mutants lacking one or another CPs (Gao *et al*., 2020) and cases when the lack of HFBs was associated with a reduced ability to attach to roots (Viterbo and Chet, 2006; Guzmán‐Guzmán *et al*., 2017). Together, this suggests that it is not EPL1 or HSF1 that prevents the attachment to roots, but rather that plants counteract the attachment process, which we explain by the actions of plant defence mechanisms. The surfaces of roots, in particular root hairs, must be suitable for the efficient absorption of water. The abundant surface‐modulating proteins secreted directly into the rhizosphere by fungi such as *Trichoderma* can alter the proper state of the root surfaces and impede plant growth. In this study, we demonstrated that the addition of recombinant saSSCPs to hydroponic medium has a significant adverse effect on plant growth. We also showed that those proteins that prevented the attachment of *T*. *guizhouense* to roots (EPL1 and HFS1) also triggered an immune response in the tomato plants, while the HFBs went unrecognized by the tomato immune system. The usual silence of these genes during submerged growth may explain the lack of a tomato response to HFBs (Cai *et al*., 2020). This fits the possibility that roots are usually confronted with CPs and HFSs but not HFBs. As a result, they may lack an efficient system for HFB recognition. Interestingly, the complete secretomes of *T*. *guizhouense* and the fungus itself were beneficial for tomato growth, likely due to the presence of such secondary metabolites as plant hormones (Vinale *et al*., 2009; Cai *et al*., 2013) and other small molecules (Harman *et al*., 2004; Li *et al*., 2015). We assume that under natural conditions, the outcome of fungal–plant interactions for the partners is determined by the trade‐offs between fungal‐beneficial and plant‐beneficial processes and that the balance is determined by the environmental conditions, which in turn, trigger the secretion of a suitable saSSCP cocktail.

We also detected a new family (HFSs) of saSSCPs with an amphiphilic nature similar to that of the HFBs and CPs. Their hydropathy profiles resemble those of relatively hydrophilic HFBs such as HFB6, HFB9a, HFB9b and HFB10. The evolutionary analysis performed for the specific HFS‐encoding genes (Fig. 3) showed that they are commonly present in all Pezizomycotina fungi genomes and have no specificity towards a particular lifestyle. The evolutionary history indicated numerous LGT events and duplications, supporting the functional necessity of these proteins in fungi. Similar results were obtained when the phylogeny of the CPs in Pezizomycotina was studied (Gao *et al*., 2020).

In conclusion, we would like to highlight that SSCPs in general, and the surface‐active members (saSSCPs) of this group in particular, are unique fungal proteins. Therefore, they should be considered from the perspective of hyphal needs rather than the response of other organisms to their presence. We propose that the saSSCPs studied here constitute a part of a larger group of hyphosphere‐specific proteins that modulate hyphal attachment to surfaces and interactions with other organisms. This work provides initial results in the investigation of a protein‐based surface recognition system in fungi that results in a sophisticated cocktail of surface‐active proteins altering the environment around the hyphae.

## Experimental procedures

### Microbial strains, plant materials and cultivation conditions

*Trichoderma guizhouense* strain NJAU 4742 (Zhang *et al*., 2016; Zhang *et al*., 2019; Cai *et al*., 2020; Gao *et al*., 2020; Pang *et al*., 2020), which is commercially available as a biofertilizer agent in China, was used as the wild type throughout this work. The sibling species *T*. *harzianum* (CBS 226.95) was also included as a reference strain when performing the colonization experiment. To express the *Trichoderma* genes in yeast, the *Pichia pastoris* strain KM71H from the EasySelect™ *Pichia* Expression Kit (Invitrogen) was used, with the plasmid pPICZαA used as the backbone vector. *Pichia pastoris* strains PpEPL1 (Gao *et al*., 2020), PpHFB2 and PpHFB4 (Cai *et al*., 2021) which produce a recombinant EPL1, HFB2 and HFB4 respectively, were generated in our previous works. If not otherwise stated, the *Trichoderma* strains were maintained on potato dextrose agar (BD Difco, USA) at 25 °C. The *Pichia* strains were maintained on yeast extract peptone dextrose agar (Sigma‐Aldrich, USA) and fermented in a buffered minimal medium containing glycerol. Protein production was induced by methanol when needed, as described previously (Zhang *et al*., 2016; Gao *et al*., 2020). All strains used in this research are listed in Supporting Information 1 Table S3. Tomato seedlings of *Solanum lycopersicum* cv. HEZUO903 were cultivated in a hydroponic system filled with a 30% Murashige and Skoog basal salt mixture medium (MS; Sigma‐Aldrich), which was maintained under controlled conditions of 25 °C with cycled illumination (light:darkness = 12 h:12 h).

### Plant growth promotion assay

The effect on plant growth from inoculating *T*. *guizhouense* NJAU 4742 and its metabolites was evaluated by adding 0.4 ml of 10^6^ ml^−1^ spores or 0.4 ml of 5‐day 30% MS (plus 1% glucose) fermenting broth (without fungal cells) to 40 ml of MS medium supplemented with 1% glucose in the hydroponic system. In this test, 3‐week‐old tomato seedlings showing equal growth were selected and treated as described, and untreated plants were used as the control. The seedlings were allowed to grow for another 3 weeks. Data regarding the plant height and fresh weight were recorded at the end of the experiment. The root growth parameters were determined with a root scanner (Epson Perfection V700 photo, Seiko Epson, Japan).

### Standard molecular techniques

The RNeasy Plant Mini Kit (Qiagen, Germany) was used to extract total RNAs from fungal or plant materials according to the manufacturer's instructions. The RevertAid™ First Strand cDNA Kit (Thermo Scientific, USA) was used to synthesize cDNAs with an oligo (dT)_18_ primer. The qPCR reactions (20 μl) were performed with 0.5 μM of each primer, 100 ng cDNA and 10 μl of iQ™ SYBR Green PCR super mix (BioRad, USA). The thermal program was implemented in a qTOWER real‐time PCR system (Jena Analytics, Germany) with a 3 min initial denaturing at 95 °C followed by 40 cycles of 15 s at 95 °C and 30 s at 60 °C. The specificity of each qPCR reaction was verified by running a melting curve from 55 to 95 °C. PCR reactions (20 μl) contained 0.5 μM of each primer, 100 ng of template DNA, 1× Phanta Max Buffer mix and 2 U Phanta Max Super‐Fidelity DNA Polymerase (Vazyme Biotech, China). The thermal cycling parameters consisted of a 3 min initial denaturing step at 95 °C; 30 cycles of 15 s at 95 °C, 15 s at 59 °C and 30 s at 72 °C; and a 5 min final extension at 72 °C. Supporting Information 1 Table S4 provides the primer information.

### Expression of *Trichoderma* genes in *Pichia pastoris*

The encoding region of the gene of interest was amplified from *T*. *guizhouense* NJAU 4742 cDNA. The signal peptides were predicted by SignalP 5.0 (http://www.cbs.dtu.dk/services/SignalP/) and excluded from the amplicons. Each amplified fragment was cloned into the pPICZαA plasmid between the *EcoR I* and *Xba I* sites. The recombinant proteins coded by this construction harbour the yeast α‐factor signal sequence at the N‐terminus and a 6× His epitope at the C‐terminus. The resulting plasmids were transformed into *P*. *pastoris* KM71H by standard electroporation transformation, and one positive transformant expressing the designated recombinant protein was chosen for all subsequent experiments. Detailed mutant construction and confirmation are given in Supporting Information 2 Figs S4 and S5. The recombinant proteins were purified as described in Przylucka *et al*. (2017b), concentrated via Vivaspin ultrafiltration devices (Sartorius, Germany) and re‐suspended in 100 mM potassium phosphate buffer (PB; pH 5.8) or milli‐Q water.

### Protein biochemical assays

Protein samples were analysed by 15% SDS‐PAGE followed by silver staining using the SilverQuest™ Silver Staining Kit (Life Technologies, Germany). The proteins separated by SDS‐PAGE were further confirmed by blotting onto an Immobilon®‐P^SQ^ Transfer Membrane (PVDF, 0.2 μm; Merck Millipore, USA) and immunologically visualized by binding with a mouse His‐tag horseradish peroxidase antibody (GenScript, China) as described by Gao *et al*. (2020). The protein concentration was quantified with the BCA protein assay kit (Thermo Fisher Scientific, USA) according to the manufacturer's instructions.

### Protein biophysical assays

The soluble state of each recombinant protein in milli‐Q water was measured by DLS in a Malvern Zetasizer Nano‐ZS (Malvern, UK) at 25 °C. Proteins were prepared at a concentration of 0.6 g L^−1^ and filtrated through a Whatman® 0.22 μm filter (Sigma‐Aldrich). The mass weight (%) diameters of the proteins were obtained from three measurements. Milli‐Q water without proteins was used as the control.

The surface activity (modification of a material's surface hydrophobicity) of the recombinant proteins was determined by WCA measurements using a Krüss EasyDrop DSA20E (Krüss GmbH, Germany). Four replicates of each surface material (glass or PET) were prepared as described by Espino‐Rammer *et al*. (2013) with small modifications. Briefly, the surface materials were trimmed to a size of 1.5 × 0.5 cm^2^ and defatted with 70% (vol./vol.) ethanol. They were then washed in sequence with 5% (wt./vol.) Triton X‐100, 100 mM Na_2_CO_3_ and deionized water, with each wash step performed for 30 min at 50 °C. The surfaces were coated with the recombinant proteins by applying a 10 μM (dissolved in PB) concentration of the protein and incubated at 25 °C for 12 h. The coated materials were washed twice with deionized water and dried before the WCA measurements.

### *In vitro* protein bioactivity assays

The *in vitro* effects of the selected saSSCPs on plant growth and immune response were determined using the hydroponic system as described above. The proteins were applied to 3‐week‐old tomato seedlings at concentrations of 0.1 and 1 μM. In the plant growth promotion test, the seedlings were allowed to grow for an additional 3 weeks, while the seedlings used for testing the immune response were sampled for RNA extraction 48 h after applying the protein. The expression of genes corresponding to different plant defence pathways was monitored with RT‐qPCR as described above. The bioactivity of each SSCP to assist *Trichoderma* (*T*. *guizhouense* NJAU 4742 and *T*. *harzianum* CBS 226.95) colonization was investigated by quantifying the *tef1* gene copy number of each strain colonized on roots and on an artificial material, i.e. glass wool (see detailed methodology in our previous work; (Gao *et al*., 2020)).

### Genome mining and evolutionary analysis

Genome mining for HFB‐encoding genes and the new SSCP family ‐ HFS‐encoding genes in the 42 *Trichoderma* whole‐genome sequenced strains (listed in Supporting Information 1 Table S2) ‐ was performed using RapidMiner (version 8.2, USA) with the HFB‐specific cysteine sequence pattern of C‐CC‐C‐C‐CC‐C or the HFS‐specific pattern of C‐CXXX‐C‐C‐C‐C‐C‐C (X represents any possible amino acid). In addition, EffHunter (Carreón‐Anguiano *et al*., 2020) was used to search for effector proteins that were then manually verified by the presence of a secretory signal peptide (SignalP 5.0). The CP sequences were obtained from our previous report (Gao *et al*., 2020). The computation of the theoretical isoelectric point (pI) and molecular weight (*M*_w_) of the SSCPs from *T*. *guizhouense* was performed using the online Compute pI/Mw tool (https://web.expasy.org/compute_pi/). The grand average of hydropathy (GRAVY) value for each protein sequence was calculated using the GRAVY Calculator (http://www.gravy-calculator.de/index.php). Detailed hydropathy profiles were calculated using https://web.expasy.org/protscale/. The possibility of a transmembrane region for each protein was checked using an online prediction program (http://www.detaibio.com/tools/index.php?r=transmembrane%2Findex). The sequences of each protein family were aligned using the MUSCLE algorithm integrated into AliView 1.23 (Edgar, 2004; Larsson, 2014). A ML phylogenetic tree was constructed for the new proteins using IQ‐TREE 1.6.12 (ultrafast bootstrap, *N* = 1000; (Nguyen *et al*., 2015)). The Bayesian Information Criterion was applied when searching for the best amino acid substitution model with ModelFinder (integrated into the IQ‐TREE program; (Kalyaanamoorthy *et al*., 2017)). An evolutionary analysis that included the NOTUNG and T‐Rex programs was performed as described in our previous works (Druzhinina *et al*., 2018; Gao *et al*., 2020). In the NOTUNG analysis, the costs of GL, GD and LGT were set at a ratio of 1:3:9, which strictly constrains the possibility of GD and LGT events.

## Statistical analysis

The relative expression fold changes of the genes of interest were calculated according to the 2^‐ΔΔCt^ method using *tef1* as the housekeeping gene (Cai *et al*., 2020; Gao *et al*., 2020). The mean and standard deviation were calculated and statistically analysed by ANOVA and Tukey multiple comparison tests at *p* < 0.05 using STATISTICA 6 software (StatSoft, USA). The bar plot, heatmap and PCA were performed with R version 3.2.2 (https://cran.r‐project.org/bin/windows/base/old/3.2.2/). Unless otherwise stated, the data regarding plant growth, root colonization and immune response were obtained from at least three biological replicates.

## Author Contributions

I.S.D., F.C. and G.B.A. conceived and designed the study. Z.Z., F.C., R.G., M.D., S.J., P.C., G.P. and G.B.A. performed the experiments. I.S.D., F.C. and Z.Z. performed the data analysis and prepared the figures. F.C. and Z.Z. performed the phylogenetic analysis with the assistance of K.C. and I.S.D. I.S.D. and FC wrote the manuscript, with comments from Q.S. and G.B.A. All authors read and approved the manuscript.

## Supporting information


**Table S1.** Small secreted cysteine‐rich protein (SSCP) sequences.
**Table S2.** Strains used in this study.
**Table S3.** Primers used in this study.
**Table S4.** Genomes used in this study.

**Fig. S1.** Western blot (WB) confirmation of the transformed *P*. *pastoris* strains producing recombinant SSCPs (HFB12 or HFS1). Dashed line highlights the aggregated rHFB12 in the sample loading well. The corresponding silver‐stained SDS‐PAGE was shown in Fig. 4. WB visualization was performed by the ONE‐HOUR Western™ Standard Kit (GenScript, China). HRP conjugated Anti‐His Tag Mouse Monoclonal antibody (Invitrogen, USA) was used at a dilution of 1:2000. PageRuler™ Prestained Protein Ladder (Fermentas, USA) was used in the gel electrophoresis.
**Fig. S2.** The hydrodynamic diameter of each recombinant protein in milli‐Q water. The mass weight (%) diameter was measured by dynamic light scatting (DLS) in a Malvern Zetasizer Nano‐ZS (Malvern, UK) at 25 °C. Proteins were prepared in a concentration of 0.6 g L^−1^. Milli‐Q water without proteins was used as the control.
**Fig. S3.** Impact of *Trichoderma guizhouense* NJAU 4742 inoculation and its secretome on plant growth. To see, whether saSSCPs impact the plant‐growth promoting effect of *T*. *guizhouense* NJAU 4742 we collected the culture filtrate (secretome, 1% Sec) corresponding to the submerged stage growth stage when most of saSSCPs were expressed (Table 2). The spore suspension of *T*. *guizhouense* NJAU 4742 (10^4^ spores ml^−1^) (Tri) and the cell‐free Sec to the roots of tomato seedlings cultivated in a hydroponic system. Values (mean ± Sd) labelled with the same letter do not statistically significantly differ (ANOVA, *p* < 0.05).
**Fig. S4.** Schematic diagram of expressing SSCP‐encoding genes under a methanol‐inducible promoter (P_AOX1_) in *P*. *pastoris*. α‐Factor, native *Saccharomyces cerevisiae* α‐factor secretion signal peptide; T_AOX1_, native transcription termination from AOX1 gene of *P*. *pastoris*; *Sh ble* cassette, *ble* gene from *Streptoalloteichus hindustanus* driving resistance to zeocin. Heterologous expression of SSCP‐encoding genes from *T*. *guizhouense* NJAU 4742 in *P*. *pastoris* strain KM71H was performed as described in Gao *et al*. (2020). The amplified gene of interest (*hfb12* and *hfs1*) (without signal peptide or intron sequences) was respectively inserted into the position between the restriction site of *EcoR I* and *Xba I* of plasmid pPICZαA. The electroporation resulted in numerous positive transformants on zeocin‐contained (100 μg ml^−1^) YPD plates (shown in Supporting Information 2 **Fig. S4** with three randomly selected mutants).
**Fig. S5.** PCR verification of *Pichia* mutants expressing *hfb12* or *hfs1*. PpVEC, represents the mutant obtained by transforming the original vector pPICZαA (without a *hfb*, *epl* or *hfs* gene) to *P*. *pastoris* cells; PpHFB12, represents mutants harbouring *hfb12*; PpHFS1, represents mutants harbouring *hfs1*. DL2000 DNA marker (Vazyme, China) was used in the gel electrophoresis. PCR products were further confirmed by sequencing.
