# Supplementary material for: At least three families of hyphosphere small secreted cysteine‐rich proteins can optimize surface properties to a moderately hydrophilic state suitable for fungal attachment
Source: Environ Microbiol. 2021 May 19;23(10):5750–68. doi: 10.1111/1462-2920.15413 (PMC8596622; doi:10.1111/1462-2920.15413)
Supplement: Supplementary file 2 — Fig. S1. Western blot (WB) confirmation of the transformed P. pastoris strains producing recombinant SSCPs (HFB12 or HFS1). Dashed line highlights the aggregated rHFB12 in the sample loading well. The corresponding silver‐stained SDS‐PAGE was shown in Fig. 4. WB visualization was performed by the ONE‐HOUR Western™ Standard Kit (GenScript, China). HRP conjugated Anti‐His Tag Mouse Monoclonal antibody (Invitrogen, USA) was used at a dilution of 1:2000. PageRuler™ Prestained Protein Ladder (Fermentas, USA) was used in the gel electrophoresis. Fig. S2. The hydrodynamic diameter of each recombinant protein in milli‐Q water. The mass weight (%) diameter was measured by dynamic light scatting (DLS) in a Malvern Zetasizer Nano‐ZS (Malvern, UK) at 25 °C. Proteins were prepared in a concentration of 0.6 g L−1. Milli‐Q water without proteins was used as the control. Fig. S3. Impact of Trichoderma guizhouense NJAU 4742 inoculation and its secretome on plant growth. To see, whether saSSCPs impact the plant‐growth promoting effect of T. guizhouense NJAU 4742 we collected the culture filtrate (secretome, 1% Sec) corresponding to the submerged stage growth stage when most of saSSCPs were expressed (Table 2). The spore suspension of T. guizhouense NJAU 4742 (104 spores ml−1) (Tri) and the cell‐free Sec to the roots of tomato seedlings cultivated in a hydroponic system. Values (mean ± Sd) labelled with the same letter do not statistically significantly differ (ANOVA, p < 0.05). Fig. S4. Schematic diagram of expressing SSCP‐encoding genes under a methanol‐inducible promoter (PAOX1) in P. pastoris. α‐Factor, native Saccharomyces cerevisiae α‐factor secretion signal peptide; TAOX1, native transcription termination from AOX1 gene of P. pastoris; Sh ble cassette, ble gene from Streptoalloteichus hindustanus driving resistance to zeocin. Heterologous expression of SSCP‐encoding genes from T. guizhouense NJAU 4742 in P. pastoris strain KM71H was performed as described in Gao et al. (2020). [file EMI-23-5750-s002.docx]

**Supplementary Information 2**

**At least three families of hyphosphere small secreted cysteine‐rich proteins can optimize surface properties to a moderately hydrophilic state suitable for fungal attachment**

Zheng Zhao^1,2^, Feng Cai^1–3*^, Renwei Gao^1,2^, Mingyue Ding^1,2^, Siqi Jiang^1,2^, Peijie Chen^1,2^, Guan Pang^1,2^, Komal Chenthamara^3^, Qirong Shen^1^, Günseli Bayram Akcapinar^4*^, Irina S. Druzhinina^1–3*^

^1^Key Laboratory of Plant Immunity, Jiangsu Provincial Key Lab of Solid Organic Waste Utilization, Nanjing Agricultural University, Nanjing, China

^2^Fungal Genomics Laboratory (FungiG), Nanjing Agricultural University, Nanjing, China

^3^Institute of Chemical, Environmental and Bioscience Engineering (ICEBE), TU Wien, Vienna, Austria

^4^Department of Medical Biotechnology, Institute of Health Sciences, Acibadem Mehmet Ali Aydinlar University, Istanbul, Turkey


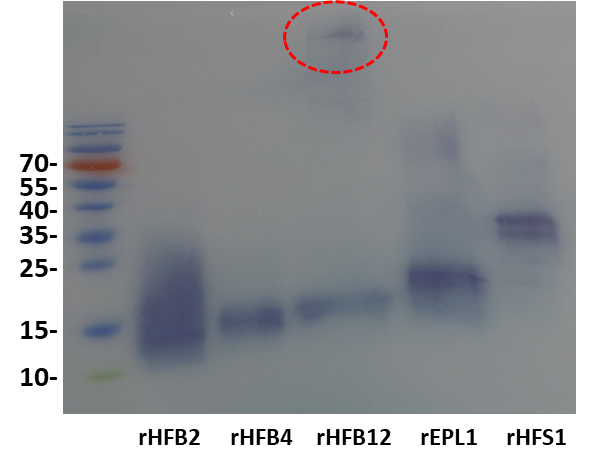


### Figure S1

Western Blot (WB) confirmation of the transformed *P. pastoris* strains producing recombinant SSCPs (HFB12 or HFS1). Dashed line highlights the aggregated rHFB12 in the sample loading well. The corresponding silver-stained SDS-PAGE was shown in **Fig. 4**. WB visualization was performed by the ONE-HOUR Western^TM^ Standard Kit (Genscript, China). HRP conjugated Anti-His Tag Mouse Monoclonal antibody (Invitrogen, USA) was used at a dilution of 1:2000. PageRuler™ Prestained Protein Ladder (Fermentas, USA) was used in the gel electrophoresis.


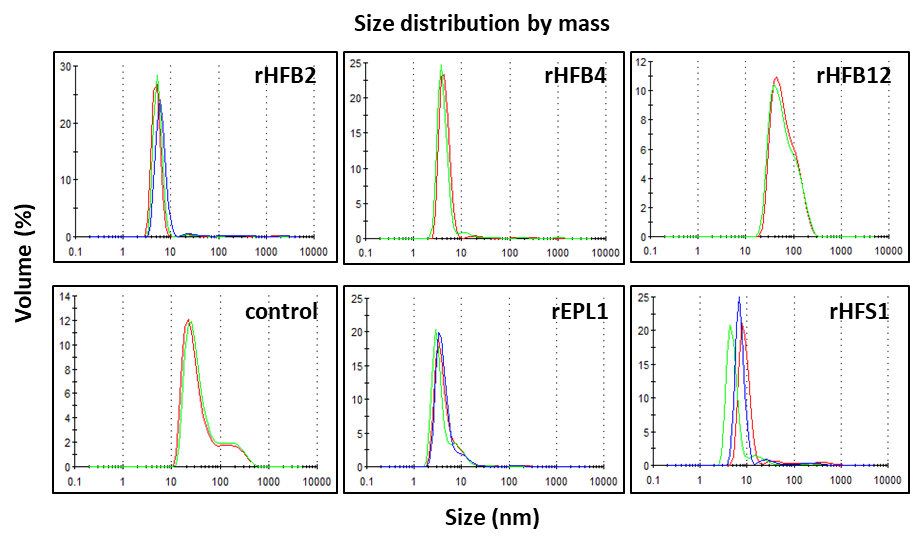


### Figure S2

The hydrodynamic diameter of each recombinant protein in milli-Q water. The mass weight (%) diameter was measured by dynamic light scatting (DLS) in a Malvern Zetasizer Nano-ZS (Malvern, UK) at 25 °C. Proteins were prepared in a concentration of 0.6 g L^-1^. Milli-Q water without proteins was used as the control.


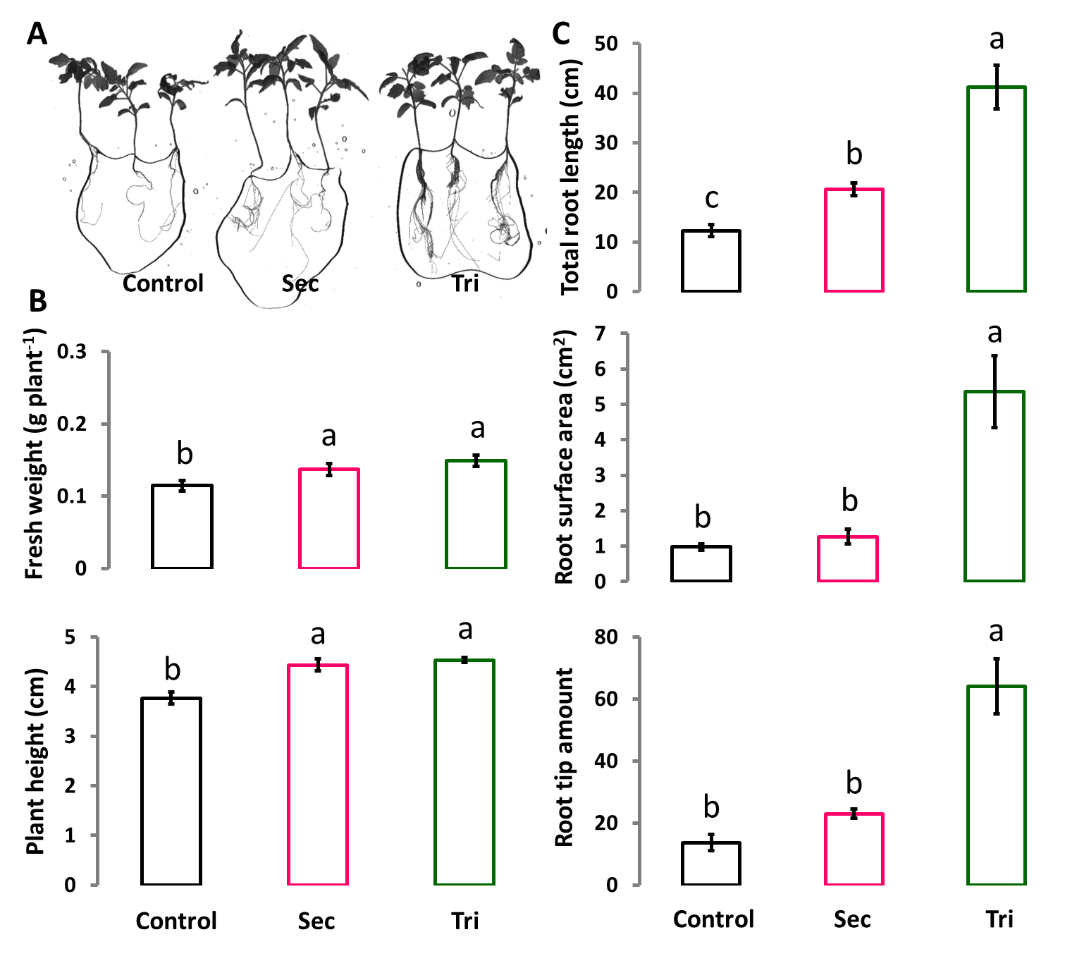


### Figure S3

Impact of *Trichoderma guizhouense* NJAU 4742 inoculation and its secretome on plant growth. To see, whether saSSCPs impact the plant-growth promoting effect of *T. guizhouense* NJAU 4742 we collected the culture filtrate (secretome, 1% Sec) corresponding to the submerged stage growth stage when most of saSSCPs were expressed (Table 2). The spore suspension of *T. guizhouense* NJAU 4742 (10^4^ spores ml^-1^) (Tri) and the cell-free Sec to the roots of tomato seedlings cultivated in a hydroponic system. The root growth parameters were determined with a root scanner (Epson perfection V700 photo, Seiko Epson, Japan). Values (mean ± Sd) labeled with the same letter do not statistically significantly differ (ANOVA, *p* >0.05).


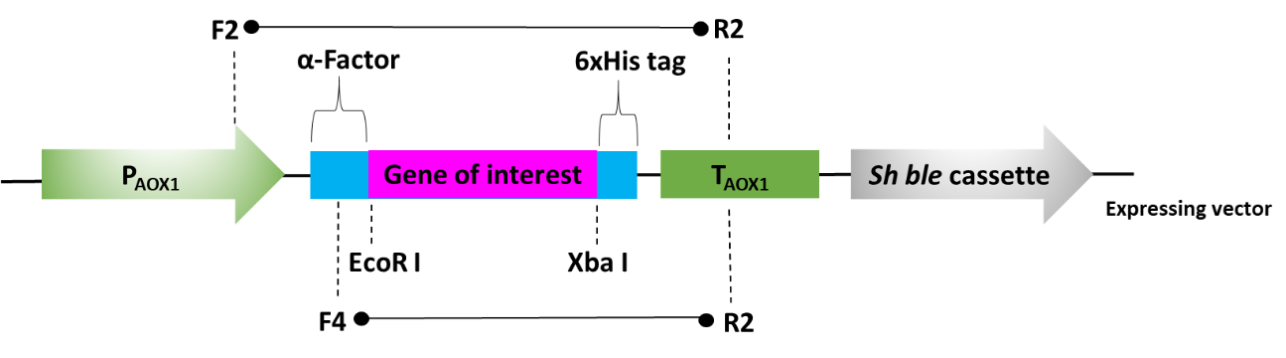


### Figure S4

Schematic diagram of expressing SSCP-encoding genes under a methanol-inducible promoter (P_AOX1_) in *P. pastoris*. α-Factor, native *Saccharomyces cerevisiae* α-factor secretion signal peptide; T_AOX1_, native transcription termination from AOX1 gene of *P. pastoris*; *Sh ble* cassette, ble gene from *Streptoalloteichus hindustanus* driving resistance to Zeocin. Heterologous expression of SSCP-encoding genes from *T. guizhouense* NJAU 4742 in *P. pastoris* strain KM71H was performed as described in Gao et al., (2020). The amplified gene of interest (*hfb12* and *hfs1*) (without signal peptide or intron sequences) was respectively inserted into the position between the restriction site of EcoR I and Xba I of plasmid pPICZαA. The electroporation resulted in numerous positive transformants on zeocin-contained (100 μg ml^-1^) YPD plates (shown in **Figure S4** with three randomly selected mutants).


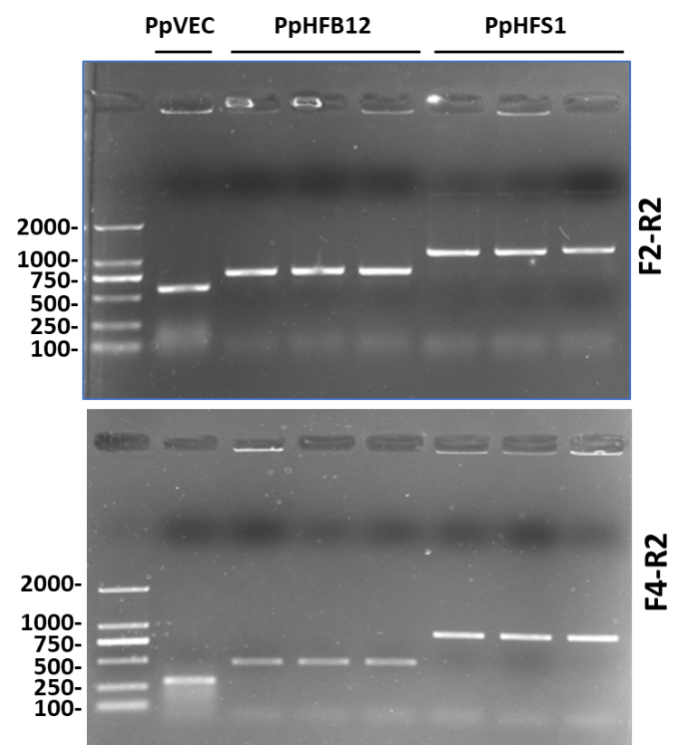


### Figure S5

PCR verification of *Pichia* mutants expressing *hfb12* or *hfs1*. PpVEC, represents the mutant obtained by transforming the original vector pPICZαA (without a *hfb*, *epl* or *hfs* gene) to *P. pastoris* cells; PpHFB12, represents mutants harboring *hfb12*; PpHFS1, represents mutants harboring *hfs1*. DL2000 DNA marker (Vazyme, China) was used in the gel electrophoresis. PCR products were further sequencing confirmed.
